# Changes in Continuous, Long-Term Heart Rate Variability and Individualized Physiological Responses to Wellness and Vacation Interventions Using a Wearable Sensor

**DOI:** 10.3389/fcvm.2020.00120

**Published:** 2020-07-31

**Authors:** Abhishek Pratap, Steve Steinhubl, Elias Chaibub Neto, Stephan W. Wegerich, Christine Tara Peterson, Lizzy Weiss, Sheila Patel, Deepak Chopra, Paul J. Mills

**Affiliations:** ^1^Sage Bionetworks, Seattle, WA, United States; ^2^Department of Biomedical Informatics and Medical Education, University of Washington, Seattle, WA, United States; ^3^Scripps Translational Science Institute, La Jolla, CA, United States; ^4^PhysIQ, Chicago, IL, United States; ^5^Department of Family Medicine and Public Health, University of California, San Diego, San Diego, CA, United States; ^6^The Chopra Foundation, Carlsbad, CA, United States; ^7^Chopra Global, New York, NY, United States

**Keywords:** remote monitoring, cardiovascular medicine, stress, wellness, digital health, wearable, intervention

## Abstract

There are many approaches to maintaining wellness, including taking a simple vacation to attending highly structured wellness retreats, which typically regulate the attendee's personal time and activities. In a healthy English-speaking cohort of 112 women and men (aged 30–80 years), this study examined the effects of participating in either a 6-days intensive wellness retreat based on Ayurvedic medicine principles or unstructured 6-days vacation at the same wellness center setting. Heart rate variability (HRV) was monitored continuously using a wearable ECG sensor patch for up to 7 days prior to, during, and 1-month following participation in the interventions. Additionally, salivary cortisol levels were assessed for all participants at multiple times during the day. Continual HRV monitoring data in the real-world setting was seen to be associated with demographic [HRV_ALF_: β_Age_ = 0.98 (95% CI = 0.96–0.98), false discovery rate (FDR) < 0.001] and physiological characteristics [HRV_PLF_: β = 0.98 (95% CI = 0.98–1), FDR =0.005] of participants. HRV features were also able to quantify known diurnal variations [HRV_LF/HF_: β_ACT:night vs. early−morning_ = 2.69 (SE = 1.26), FDR < 0.001] along with notable inter- and intraperson heterogeneity in response to intervention. A statistically significant increase in HRV_ALF_ [β = 1.48 (SE = 1.1), FDR < 0.001] was observed for all participants during the resort visit. Personalized HRV analysis at an individual level showed a distinct individualized response to intervention, further supporting the utility of using continuous real-world tracking of HRV at an individual level to objectively measure responses to potentially stressful or relaxing settings.

## Introduction

Stress, in varying degrees and of varying durations, impacts every single human and has a major influence on their health (1). Healthy individuals typically can manage short, acute episodes of routine stress without health consequences. However, if stress becomes persistent, the long-term effects can significantly impair mental and physiological well-being (2). When recognized, people can often address and ideally eliminate specific acute stressors (3). Chronic stress, however, is often insidious and develops slowly, making it difficult for an individual experiencing it to recognize it (4). In addition, the marked variability in stress responses among individuals can make it difficult to be clearly recognized by others, including family and even mental health professionals (5). Therefore, there is a critical need for objective, reproducible measures of an individual's ability to recover from stress.

The autonomic nervous system (ANS) plays an important role in the body's response to stress. Composed of the parasympathetic and sympathetic nervous systems (PNS and SNS) (1), the response to stress is driven primarily by the SNS and the hypothalamic–pituitary–adrenocortical axis (1, 6). On the other hand, the PNS is important in alleviating the stress response (7). While at present there is no universally accepted standard for objective stress measurement (8), fluctuations in the length of heartbeat intervals, commonly referred to as heart rate variability (HRV) (6, 9, 10), which is under the control of the ANS, has been found in a number of studies to be a potentially viable objective biomarker of the stress response (6). HRV also has the advantage of being non-invasively and potentially continuously measured, typically using an electrocardiogram.

Historically, one of the challenges to demonstrating the effectiveness of wellness interventions has been a focus on a population-wide effect that is based on a single “snapshot” recording taken before and after the intervention, typically in an artificial setting outside of routine daily activities. This approach, however, is often hampered by the wide interindividual variability in physiological parameters at baseline and in responsiveness to the intervention, as well as intraindividual variability that is common throughout the day. The noise created by these multiple sources of variation can easily overwhelm any metric of change that is population based and prevents identifying potentially important changes in an individual. However, with the recent growth in consumer-grade devices and wearables (11, 12), new and innovative ways of collecting real-world (13) multi physiological data for prolonged periods of time have become feasible (14, 15). This rich data allow for a detailed and personalized analysis of normal physiological changes and how they relate to each other, over time, and in the daily lives of people. Such a multivariate longitudinal measure of an individual's physiology can enable assessment of short- and long-term changes in the functioning of the autonomous nervous system (16, 17) especially relative to one's own baseline. In particular, features related to HRV, generated using biosignals obtained from continual monitoring of heart, have been linked with ANS-related neurocardiac functioning such as stress, autonomic balance, blood pressure (BP), and vascular tone including being impacted by external environmental and psychological variations over time (18, 19). To quantify such complex and dynamic non-linear associations between ANS functioning and HRV, it is recommended to use both the time and frequency domain features of HRV (10, 20). Briefly, time-domain HRV features (21, 22) quantify the variation in the interbeat interval (IBI) between successive heartbeats with standard deviation of the IBI of normal sinus beats [standard deviation of interbeat intervals (SDNN)] considered the gold standard for clinical cardiac risk evaluation (23). The frequency- and amplitude-based HRV features are derived based on power spectral analysis (24) to derive energy within a prespecified four frequency bands (23). Of these, the low-frequency (LF) (0.04–0.15 Hz, between 7 and 25 s) band has been linked to vagal and sympathetic activity, whereas the high-frequency (0.15–0.4 Hz, between 2.5 and 7 s) has been linked to parasympathetic activity of the ANS (20). Salivary cortisol is another frequently used biomarker of stress as a measure of hypothalamus–pituitary–adrenal axis (HPAA) activity. Obtaining repeated saliva samples during the course of a day is an established method of determining the diurnal variation of cortisol, including for intervention studies (25, 26).

In the current study, utilizing a novel sensor patch that could monitor multiple physiological parameters, including ECG, continuously for up to 7 days, we investigated whether it was feasible to detect changes in HRV relative to a participant's baseline, in response to two different week-long wellness programs, one a structured wellness program and the other an unstructured wellness vacation at the same resort location (27). We further explored if changes in HRV sustained over a period of time by remotely monitoring participants 1 month after the interventions. Additionally, any significant differences in salivary cortisol levels across home, wellness center visit, and across the two cohorts were also assessed for their relationship to HRV.

## Methods

### Study Participants and Design

**Figure 1:**
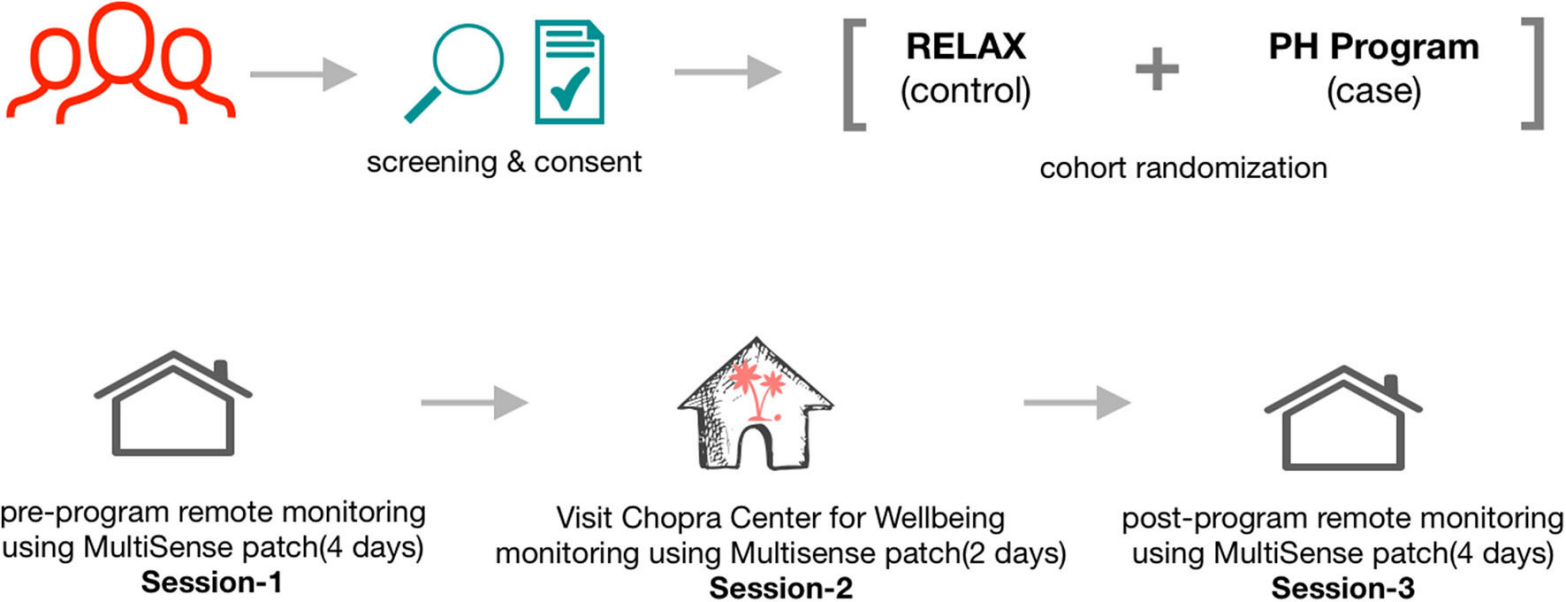
Overall schematic flow of the study showing participant recruitment, screening, consent, and wearable sensor-based tracking of physiological characteristics across the three sessions. The week prior to starting the program (session 1), the last 2 days of the respective intervention program (session 2), and at home ~1 month after completion of the intervention programs (session 3).

Participants were recruited via email announcements from the UC San Diego faculty and staff and Chopra Center for Well-Being mailing lists. The announcements described the Self-Directed Biological Transformation Initiative (SBTI), a research study examining the effects of a structured wellness program based on Ayurvedic principles called the Perfect Health Program (PH) or an unstructured vacation retreat (RELAX) (28). The majority of enrolled participants were from the greater San Diego and Los Angeles areas. Flyers stated that the week-long (28) study would be conducted at the La Costa Resort in Carlsbad, CA and that participants would either participate in the PH program or take a regular vacation at the same resort. All study participants stayed onsite at the resort for 1 week (Figure 1). The study was approved by the UC San Diego Institutional Review Board (#171715) and was registered on ClinicalTrials.gov with Identifier: NCT02241226.

Eligible participants were healthy English-speaking women and men aged 30–80 years with no current major medical or mental illnesses. Exclusion criteria included being a smoker, pregnancy, estrogen or hormone replacement therapy, a body mass index (BMI) ≥35 kg/m^2^, and current use of non-prescription drugs. Individuals were not excluded if they took prescription drugs. Participants were willing to refrain from drinking more than one alcoholic beverage per day during the weeklong stay at the resort. Finally, individuals who have previously participated in the PH program or other Chopra Center programs or a yoga or meditation retreat of any kind within the past 12 months were also excluded from study participation.

A total of 261 individuals were screened for eligibility, learned details of study involvement, and were asked to consider their commitment and availability before they were enrolled in the study. It was emphasized that this included a commitment to stay in the study even if they were not randomized into their preferred group. A total of 112 individuals consented to enroll in the study and were randomly assigned in a 1:2 ratio to either the PH group or the RELAX group. Within the enrolled cohort, a group of 23 individuals (20.5%) who were previously planning to take the PH program were assigned to the PH program in a non-randomized fashion. There were no expenses for participation in the study. Participants were not compensated financially or with any type of non-financial incentives other than having their expenses covered while at the resort.

### Study Interventions

The intervention groups were not blinded, and site investigators and study personnel knew to which group participants were assigned. The two groups had no contact with each other during the study and did not know the details of the daily schedule of the other group. Upon arrival at the resort, participants were given a 1-h orientation meeting with the study team where they learned about the overall study schedule and procedures and the assessment schedules.

### PH Program Intervention

PH is essentially an Ayurvedic immersion program of detoxification and rejuvenation that is based on core principles from the Ayurvedic system of medicine (28). The program was developed ~20 years ago, with ~800 individuals taking the course every year. The PH program addresses the physical, emotional, and spiritual well-being through daily practices and lectures. Components of the program included physical cleansing through ingestion of herbs, fiber, and oils, two Ayurvedic meals daily (breakfast and lunch), which provided a light plant-based diet, daily Ayurvedic massage treatments, and heating treatments through the use of dry sauna and/or steam sauna. The program includes lectures on Ayurvedic principles and lifestyle as well as lectures on meditation and yoga philosophy. The PH study group participated in twice-daily group meditation and daily yoga, practiced breathing exercises, as well as emotional expression through a process of journaling and emotional support. The daily yoga practice consisted of a standard set of 10 yoga postures. During the program, PH participants received a 1-h integrative medical consultation with a physician and a follow-up with an Ayurvedic health educator. The teachers of the PH Program delivered their standard program to the SBTI study participants.

### RELAX Intervention

Participants randomized to the RELAX group were asked to do what they would normally do on a resort vacation with the additional following restrictions: they were asked not to engage in more exercise than they would in their normal lifestyle and to refrain from using La Costa Resort spa services. RELAX participants were also asked not to drink ginger tea or take gingko biloba during the 2 days prior to and during the study week.

### Salivary Cortisol

To assess diurnal variations in cortisol levels, saliva samples at four different periods were collected: at the participant's home the week prior to coming to the retreat, day 1 of the retreat, day 6 of the retreat, and at home 1 month following the retreat. Participants were provided an information sheet on how to properly self-collect, label, and store their saliva samples. Participants collected their saliva into salimetrics saliva collection tubes using the passive drool method at the following times: immediately upon awakening, 30 min post-awakening, noon, 4:30 pm, 8:00 pm, and bedtime. Upon collection, participants put their samples on ice or in a −20°C freezer. For samples collected at home, participants placed the samples on ice packs and sent them to the UC San Diego Clinical Research Biomarker Laboratory samples using overnight shipping, where they were placed in −80°C until assay (29). Saliva cortisol levels were determined by commercial ELISA (Salimetrics, Carlsbad, CA, USA). Intraassay coefficients of variation were <5%; interassay coefficients of variation were <8%.

### Continual Physiological Monitoring

**Figure 2:**
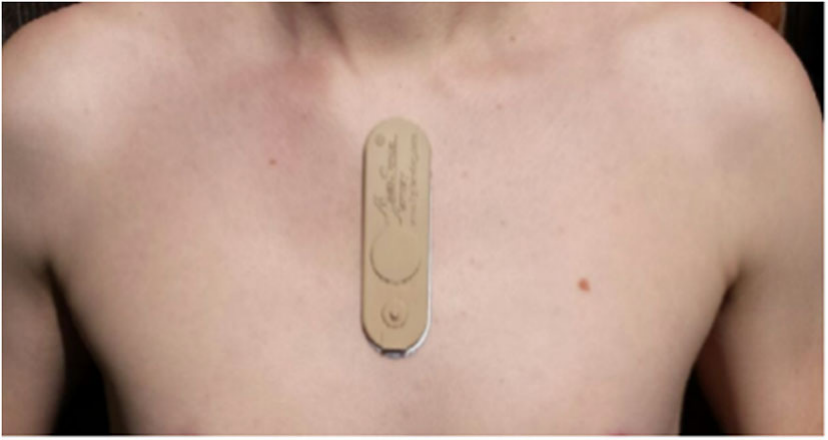
Picture of the Rhythm Diagnostic Systems (RDS) patch and recommended placement on the body.

An adhesive wearable patch (MultiSense™) developed by Rhythm Diagnostic Systems (RDS), which has multiple sensors embedded in a “Band-Aid”-like strip (Figure 2), was used to continually monitor multiple physiological parameters of study participants. MultiSense™ wearable patch is a self-contained, reusable, rechargeable, battery-powered, flexible strip, measuring 4 × 1.2 in and weighs <15 g. The MultiSense device collects and stores raw biosensor data from a single lead ECG, a three-axis accelerometer, a skin temperature sensor, and a Red and IR photoplethysmography (PPG) sensor. The device stores all physiological data in onboard memory and can be worn for up to 10 days with the data then downloaded via USB.

All study participants were asked to wear the sensor patch on three occasions: the week prior to starting the program (session 1), the last 2 days of the program (session 2), and at home ~1 month after completion of the program (session 3). The wearable sensor was one of the many components of a much broader SBTI program (28, 30).

### Feature Extraction

**Figure 3:**
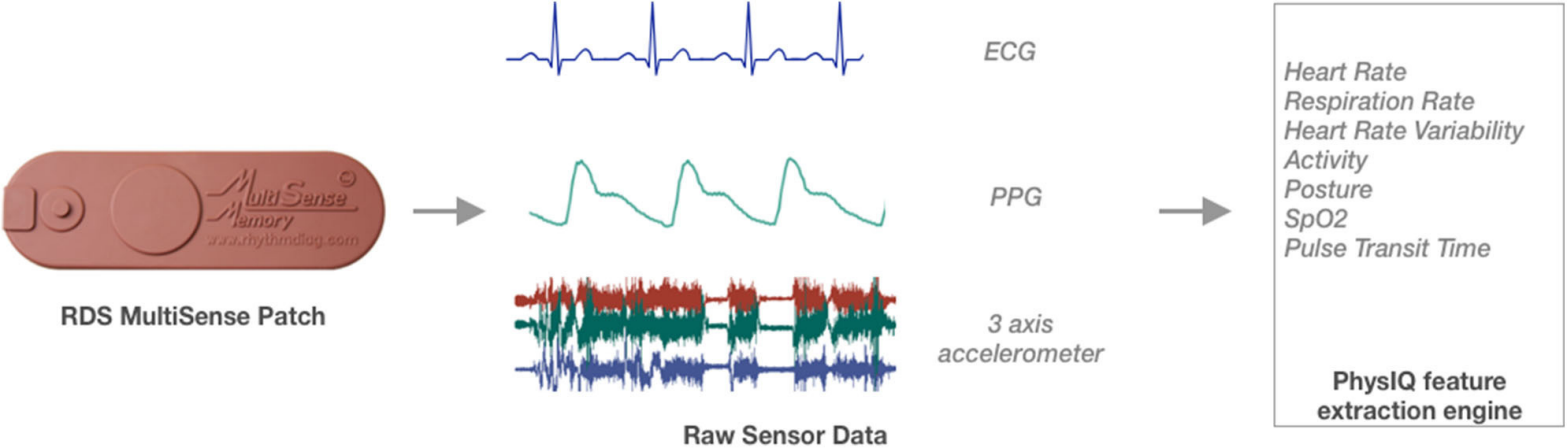
Schematic overview of feature extraction translating the raw sensor data collected using the Rhythm Diagnostic Systems (RDS) MultiSense patch.

Vital signs and physiological features were derived from the raw sensor data using a proprietary library of signal processing and data analytics algorithms developed by PhysIQ (31) (Figure 3). The offline feature extraction engine, using the raw sensor data [ECG, a three-axis accelerometer, PPG (red & IR)], generated 22 cardiopulmonary-related features. For the present analyses, we used a subset of eight heart rate and HRV-related features (Table 1) that are known to be a potential biomarker for stress (6, 32).

**Table 1:** Brief description of features generated by the feature extraction engine that were used for the present analysis.

**Vital sign label**	**Feature domain**	**Units**	**Description**
HR	Time	bpm	1-min trimmed mean time averages of beat-to-beat HR
SDNN	Time	Milliseconds	1-min trimmed mean time averages of the trimmed (remove upper and lower 5% of samples) standard deviation of interbeat intervals (SDNN) in 30-s windows calculated every 5 s
ACT	Frequency	g's	1-min trimmed mean time averages of gross activity level (essentially overall movement in all directions combined)
ALF	Frequency	s^2^	1-min trimmed mean time averages of power in the low-frequency band of heart rate variability (0.040–0.150 Hz)
AHF	Frequency	s^2^	1-min trimmed mean time averages of power in the high-frequency band of heart rate variability (0.150–0.400 Hz)
PHF	Frequency	s^2^	1-min trimmed mean time averages of normalized power in the low-frequency band of heart rate variability (0.040–0.150 Hz)
PLF	Frequency	s^2^	1-min trimmed mean time averages of normalized power in the high-frequency band of heart rate variability
LF/HF	Frequency	–	1-min trimmed mean time averages of the ratio of low frequency to high-frequency HRV power
ECG goodness	Time	%	1-min trimmed mean time averages of ECG signal quality metric (100% is good, 0% is bad)

In addition, an ECG signal quality metric was calculated that is designed to identify snippets of ECG data that can result in inaccurate feature calculations [due to loss of signal, low signal-to-noise ratio (SNR), and motion artifacts). The algorithm is based on a combination of analyzing the kurtosis of 15-s moving windows of ECG data and heuristics rules that identify non-varying samples as well as completely random samples within each window. A binary decision is rendered for each 15-s window identifying it as either “usable” or “non-usable” in the context of the feature extraction engine. A decision is made every 5 s by sliding the window of ECG data by 10 s. A corresponding proportion is generated for each minute based on the 5-s positive decisions made during each window of data. This proportion is the ECG signal quality metric. A default threshold of 0.9 (90%) is used as a basis for feature calculation quality filtering. A filtered sample is replaced with a NaN (not-a-number) value, indicating that ECG data existed during the corresponding minute, but the calculated feature is very likely unreliable.

### Statistical Analysis

The evaluation of inter- and intraindividual changes in HRV overtime was a secondary and exploratory goal of the SBTI trial, and therefore, the randomization of participants into RELAX and PH interventions was not optimally powered to assess the difference between the interventions.

Prior to analysis, the data in the upper and lower 1% quantile of HR and HRV features were filtered out to exclude potential outliers in the data due to technical noise at the cohort level. Additionally, all data points identified as likely to be indicative of poor accuracy due to biosignal noise, and the presence of motion artifacts was removed from the downstream analysis. Those samples included any with a corresponding ECG signal quality metric that was <90%. Finally, only those participants who contributed at least 24 h worth of continual sensing data were considered for the cohort-level analysis. Differences in demographics variables across the RELAX and PH groups were assessed using a chi-square test.

Generalized estimating equations (GEEs) (33) were used to estimate the population-level average effect of heart rate variability between (a) PH and RELAX cohorts, (b) three sessions (session 1: at home before the interventions; session 2: at the resort; session 3: at home 1 month after the respective intervention), and (c) diurnal variations of HRV. Briefly, a GEE is a semiparametric method to estimate population-averaged effects by accounting for time-invariant, unobservable differences within individuals using robust and unbiased standard errors. The GEE model accounted for covariates such as age, BMI, gender, and physical activity (estimated by the triaxial acceleration sensor onboard the MultiSense™ wearable patch; see Table 1). A tri-level interaction between the three covariates, cohort (RELAX and PH), three sessions, and diurnal variations, were used in the model to estimate the population level significance of these interactions on HRV. We also tried to account for the subject level heterogeneity using a linear mixed-effects (LME) modeling approach (34, 35). Several LME models were fit using the lmer (36) package with combinations of fixed and random effects; however, due to missing sensor data across the sessions for some subjects, most LME models did not converge and therefore were dropped from further analysis.

To further investigate the high interperson heterogeneity in HRV, a personalized “*N*-of-1” analysis approach was used to assess the significance of a visit to the resort (session 2) on HRV regardless of the intervention group that an individual was assigned. Newey–West robust regression (37) method was used to account for the serial correlation structure in the data. Briefly, this approach allows conducting robust regression modeling using a non-parametric kernel-based heteroskedasticity and autocorrelation consistent (HAC) estimator of the covariance matrix that is able to account for both heteroscedasticity and autocorrelation of unknown form. We further adopted the Newey–West HAC estimator, using Bartlett kernel, and the automatic bandwidth selection procedure (38), and implemented in the sandwich R package (39). Time-of-the-day and average acceleration were included as covariates to account for individual diurnal variations. All analysis was done using the R statistical programming language (40).

## Results

### Data Summary

**Figure 4:**
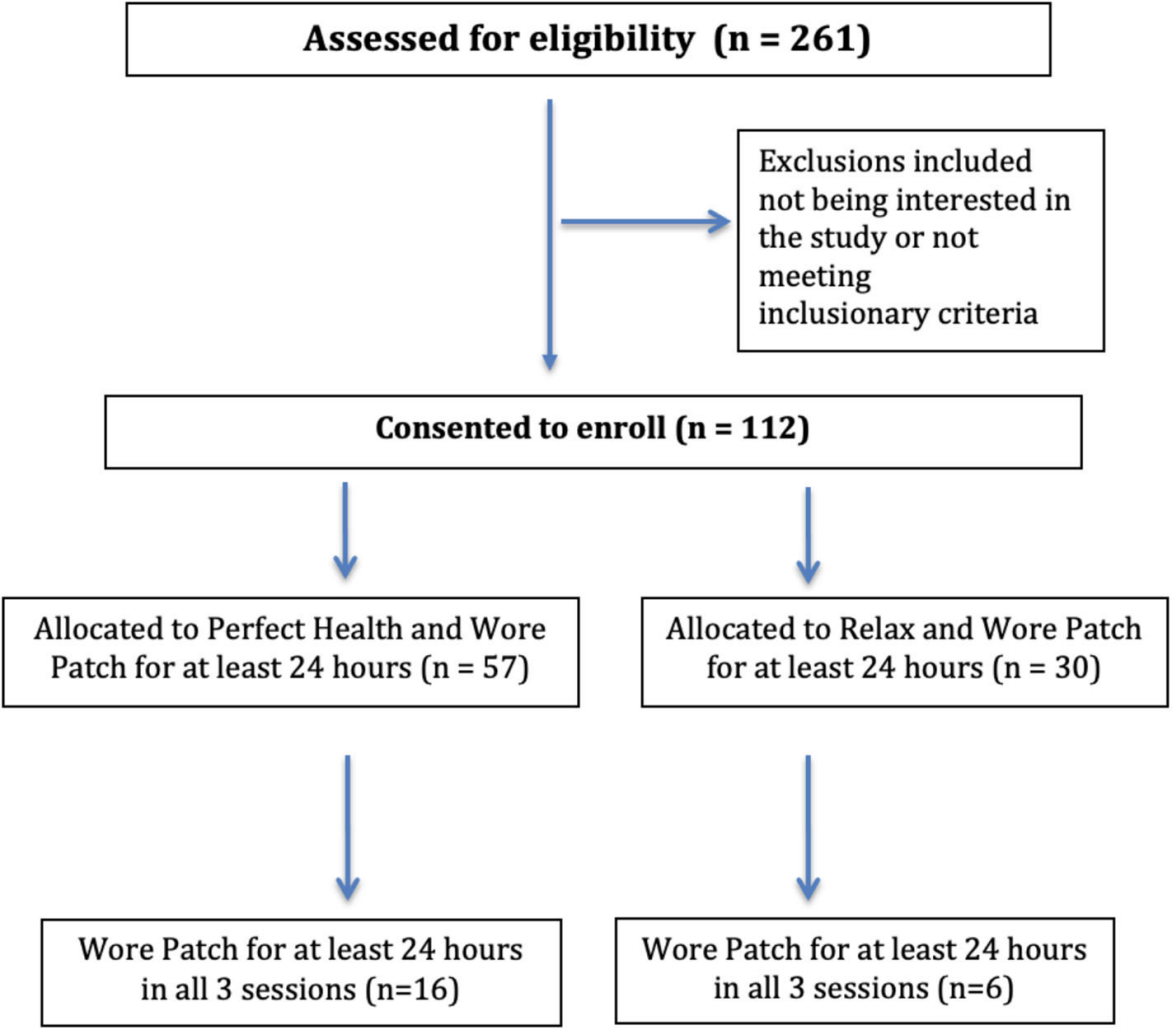
CONSORT flow diagram for Self-Directed Biological Transformation Initiative (SBTI) study participants.

A total of 261 individuals were screened for the study of which 112 individuals (69 PH, 44 RELAX) were enrolled, contributing 11,355 h worth of continual sensor-based monitoring data using the RDS MultiSense™ patch (Figure 4). Of these, 87 participants (57 PH, 30 RELAX) wore the patch and contributed at least 24 h of physiological monitoring data and were included in the population-level analysis. A smaller subset of the sample (*N* = 43) contributed at least 24 h of data for two out of the three sessions, and finally, 22 (16 PH, 6 RELAX) participants contributed at least 24 h of data for all three sessions (Supplementary Figure 1). Overall, no significant difference in baseline demographic characteristics was seen between the participants in PH and RELAX cohorts including the subset of participants that were non-randomly assigned to PH intervention (Supplementary Table 1). The mean age of the participants in the PH cohort was 53.9 (SD = 10.96) years and 56.1 (SD = 10.02) years in the RELAX cohort. The majority of participants in both cohorts were female (PH, 78.2%; RELAX, 71.4%) (Table 2).

**Table 2:** Comparison of baseline characteristics across the RELAX and PH study groups.

	**PH**	**RELAX**
*N*	57	30
Age [mean (SD)]	53.87 (10.96)	56.15 (10.02)
Gender = female (%)	43 (78.2)	20 (71.4)
Height [mean (SD)]	66.34 (3.41)	66.13 (4.19)
Weight [mean (SD)]	150.09 (31.26)	155.19 (41.45)
**Hours of MultiSense patch recording**		
Session 1 [mean (SD)]	80.18 (37.92)	59.35 (32.24)
Session 2 [mean (SD)]	39.38 (9.17)	36.97 (29.38)
Session 3 [mean (SD)]	80.37 (32.11)	72.53 (28.24)

**Figure 5:**
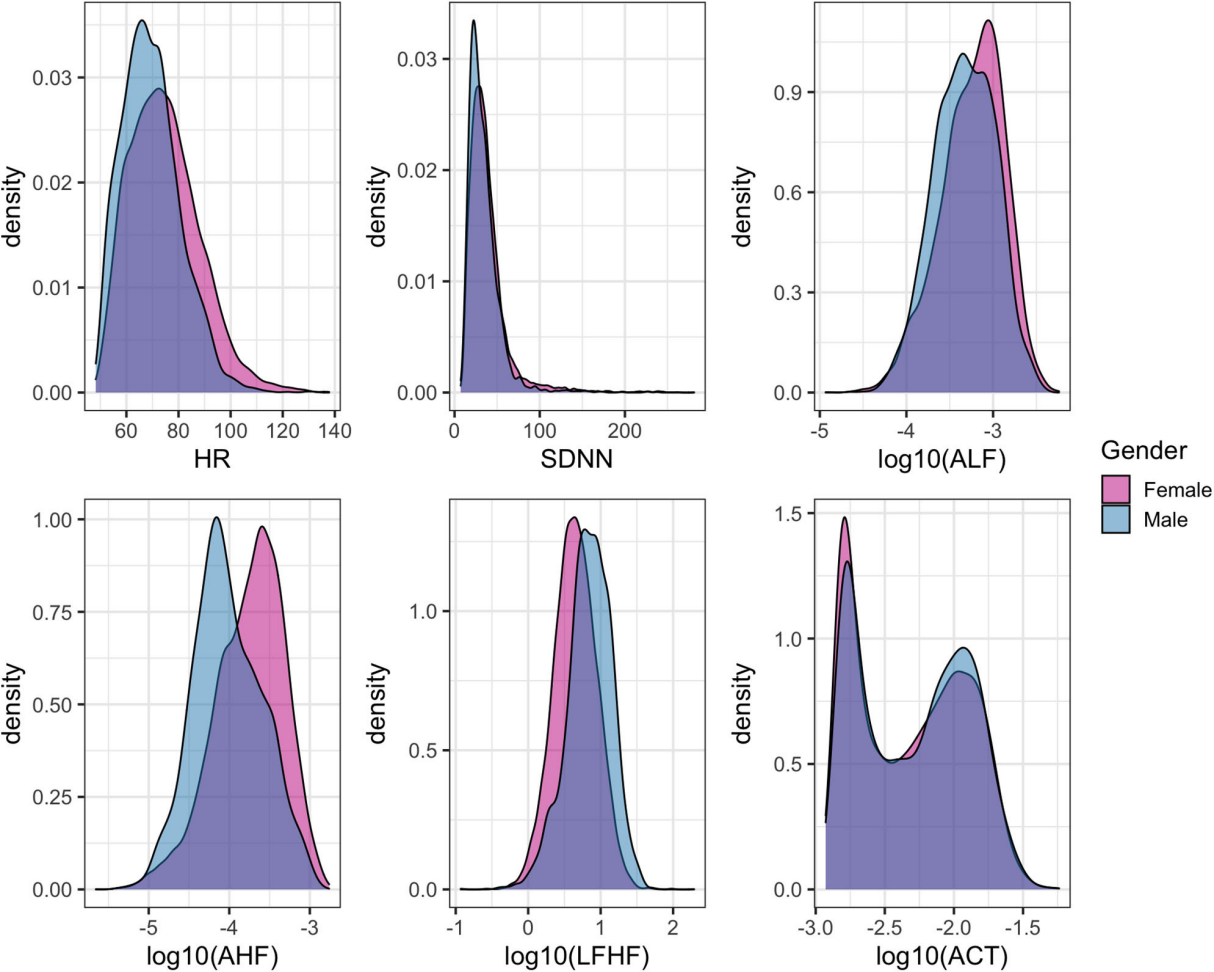
Density histograms of heart rate (HR), select features related to heart rate variability (HRV), and overall physical activity stratified by gender.

The three primary physiological parameters of heart rate, heart rate variability, and average accelerations showed similar marginal distributions across the three sessions (Figure 5). The average heart rate was 72.5 (SD = 13.6) beats per minute (bpm) with significant differences across gender (female = 73.7 ± 14.0 bpm, male = 68.7 ± 11.7 bpm, *p* < 0.0001, Figure 5). The mean HRV for the cohort was 37.7 (SD = 24.2) ms with a significant difference between gender (mean female = 38.9, male = 33.8, *p* < 0.0001).

### Cohort-Level Assessment of HRV and Salivary Cortisol Levels

The cohort level model showed a limited statistically significant impact of wellness center visit on the study participants assessed by evaluating differences in HR- and HRV-related features (Supplementary Figure 2). The topmost HRV features associated with the covariates of interest are presented here with the complete results from GEE models available as part of the Supplementary Table 2. At the population level, participant's age was statistically significantly associated with HRV feature ALF [β = 0.98 (CI = 0.96–0.98), false discovery rate (FDR) < 0.001]. Participants' BMI was also found to be statistically significantly associated the most with HRV feature PLF [β = 0.98 (CI = 0.98–1), FDR = 0.005). The high-frequency biosignals were also sensitive to capture the diurnal variations and the impact of physical activity on HR and HRV. For example, the daily variation in heart rate was significantly associated with the time of day [β_evening vs. earlymorning_= 29.60 (CI = 13.35–45.85), FDR < 0.001]. HRV-related feature LF/HF ratio was also associated jointly with the time of day and physical activity [β_ACT:night vs. earlymorning_= 2.69 (CI = 1.7–4.27), FDR < 0.001]. A statistically significant interaction between the wellness center visit and time of day was also found. All participants regardless of the subcohort (PH, RELAX) showed a significant difference in HR- or HRV-related features during session 2 (wellness center visit) in the morning and afternoon period compared to session 1 (at home). Specifically, a marginally significant decrease in heart rate was seen during the afternoon for all participants [β = −4.26 (CI = −7.58—0.94), FDR = 0.03], and HRV feature ALF showed statistically significant increase during the morning [β= 1.48 (CI = 1.23–1.82), FDR < 0.001] and afternoon [β= 1.55 (CI = 1.23–1.95), FDR = 0.001] during the wellness center visit. While a marginally statistically significant difference in one HRV-related feature PLF was observed between PH and RELAX cohort [β= 0.66 (CI = 0.49–0.89), FDR = 0.04], the change was not persistent over time (session 3). Additionally, while the salivary cortisol assay was sensitive to capturing the known diurnal variations (41, 42) in cortisol levels, no other significant differences in both PH and RELAX cohorts based on wellness center visits were observed (Figure 7, Supplementary Table 3).

### Personalized *N*-of-1 Analysis

**Figure 6:**
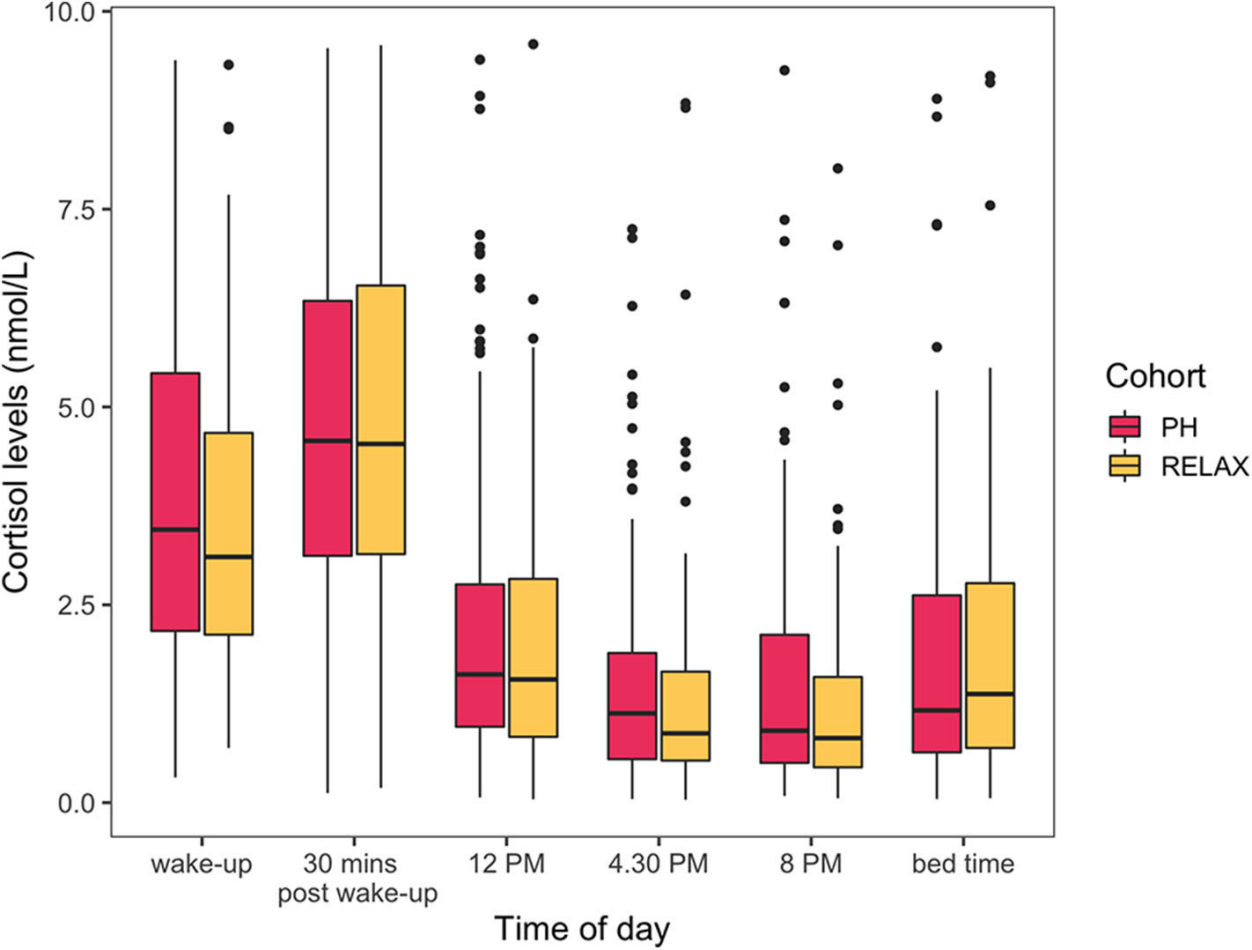
Cortisol levels (nmol/L) across the six different times of the day. Cohort level model shows no statistically significant differences in cortisol levels between RELAX and PH cohort.

With a high level inter- and intraperson variability in HRV features, personalized *N*-of-1 models for a subset of individuals (*N* = 22) that contributed data for at least 24 h across all three sessions were evaluated. Figure 6 summarizes the HRV variation at an individual level along with deviation from the median HRV of the cohort for each session. Personalized models showed statistically significant differences in at least one of the features related to HRV for 45.5% (*N* = 10) individuals in session 1 vs. 2 (home vs. wellness center visit). However, a significantly larger proportion of the participants 81.8% (*N* = 18) showed a statistically significant difference in session 1 vs. 3 (pre- vs. post-visit home) in at least one of the HRV-related features. Figure 8 shows the *p*-values (FDR corrected) obtained from the robust Newey–West regression of the pairwise differences in HRV-related features across the three sessions showing varying response patterns across participants. The results show a highly individualized response to wellness center visits. Across the heterogeneous patterns of response (Figure 8), we observed four broad profiles of participant response: (1) no statistically significant difference in HRV across the three sessions; (2) long-term responder where participant showed a statistically change in HRV during the wellness center visit (session 2) compared to the at-home assessment before wellness center visit (session 1) and persistence of change on participants' return to home (session 3) after wellness center visit; (3) short-term responder showing an increased HRV in session 2;however, it regressed toward the baseline mean in session 3; and finally, (4) decreased in HRV in session 2 that persists in the session 3 (Figure 9). Significant intraperson variability in cortisol levels in the morning time [interquartile range (IQR) = 3.1–33] was seen compared to afternoon and evening (IQR = 1.1–1.8) (Supplementary Figure 3). However, no statistical test was used to assess *N*-of-1 differences in cortisol levels due to the limited number of data points per participant (Supplementary Figure 4).

**Figure 7:**
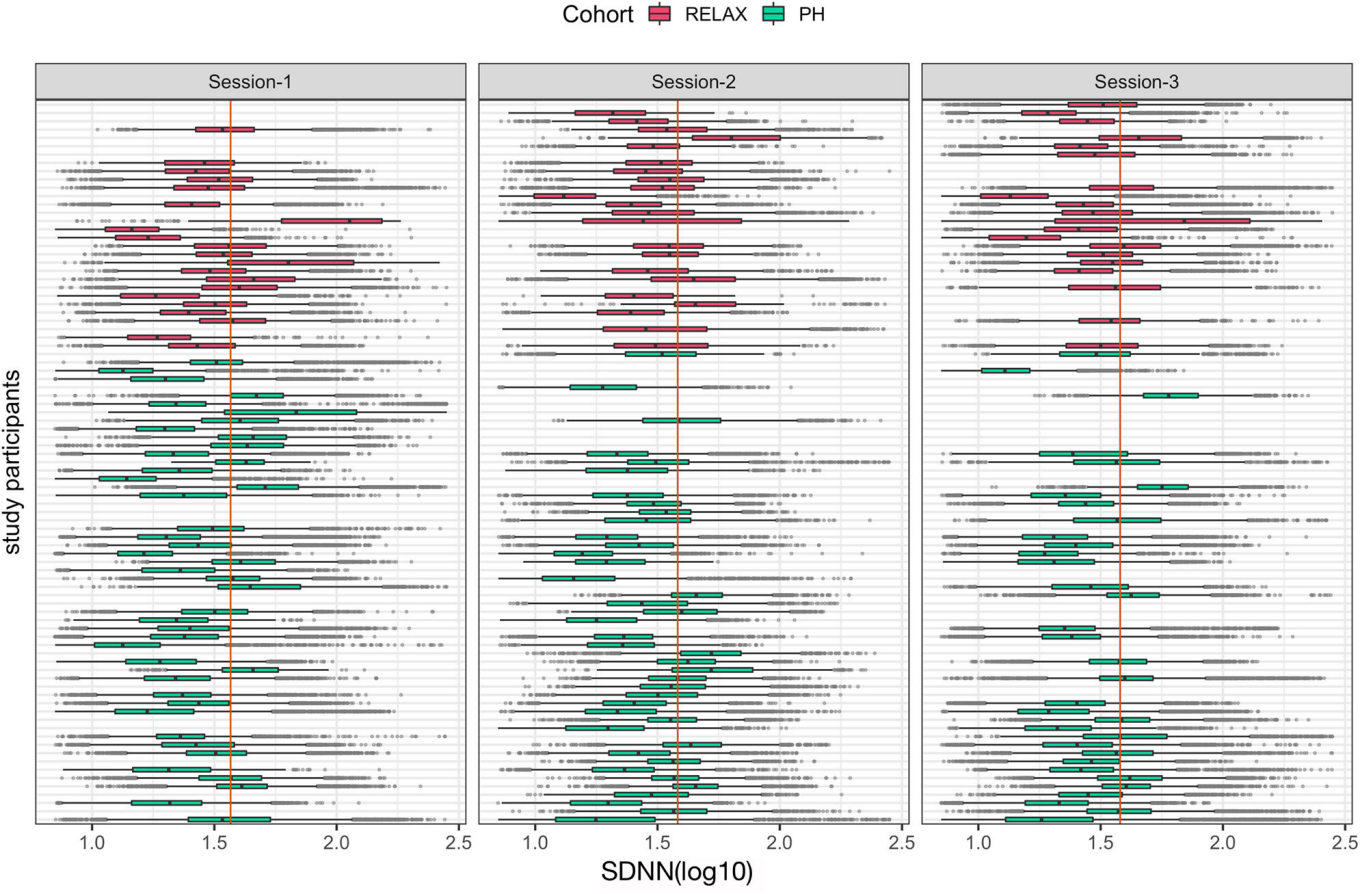
Boxplots of individual-level mean hourly heart rate variability feature, SDNN (log_10_) across the three study sessions colored by participants' cohort status. The vertical orange line in each session shows the median value of the cohort for each session.

**Figure 8:**
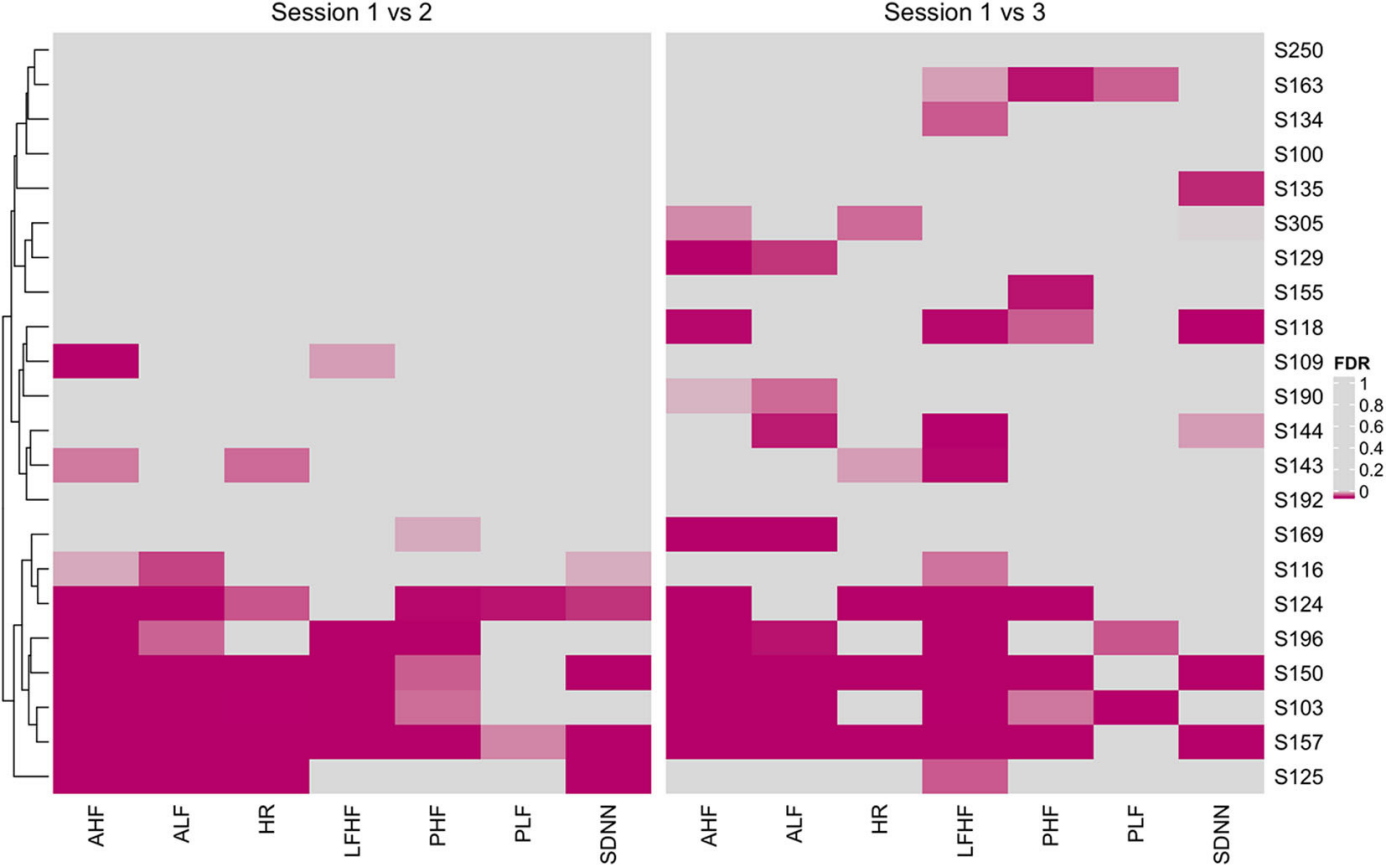
Heatmap of false discovery rate (FDR) corrected *p*-values from personalized models comparing heart rate variability (HRV)-related features across session 1 vs. 2 (home vs. wellness center visit) and session 1 vs. 3 (pre vs. post visit at home). Significantly associated HRV features (FDR < 0.05) across individuals are shown using magenta color with darker hue showing stronger significance.

**Figure 9:**
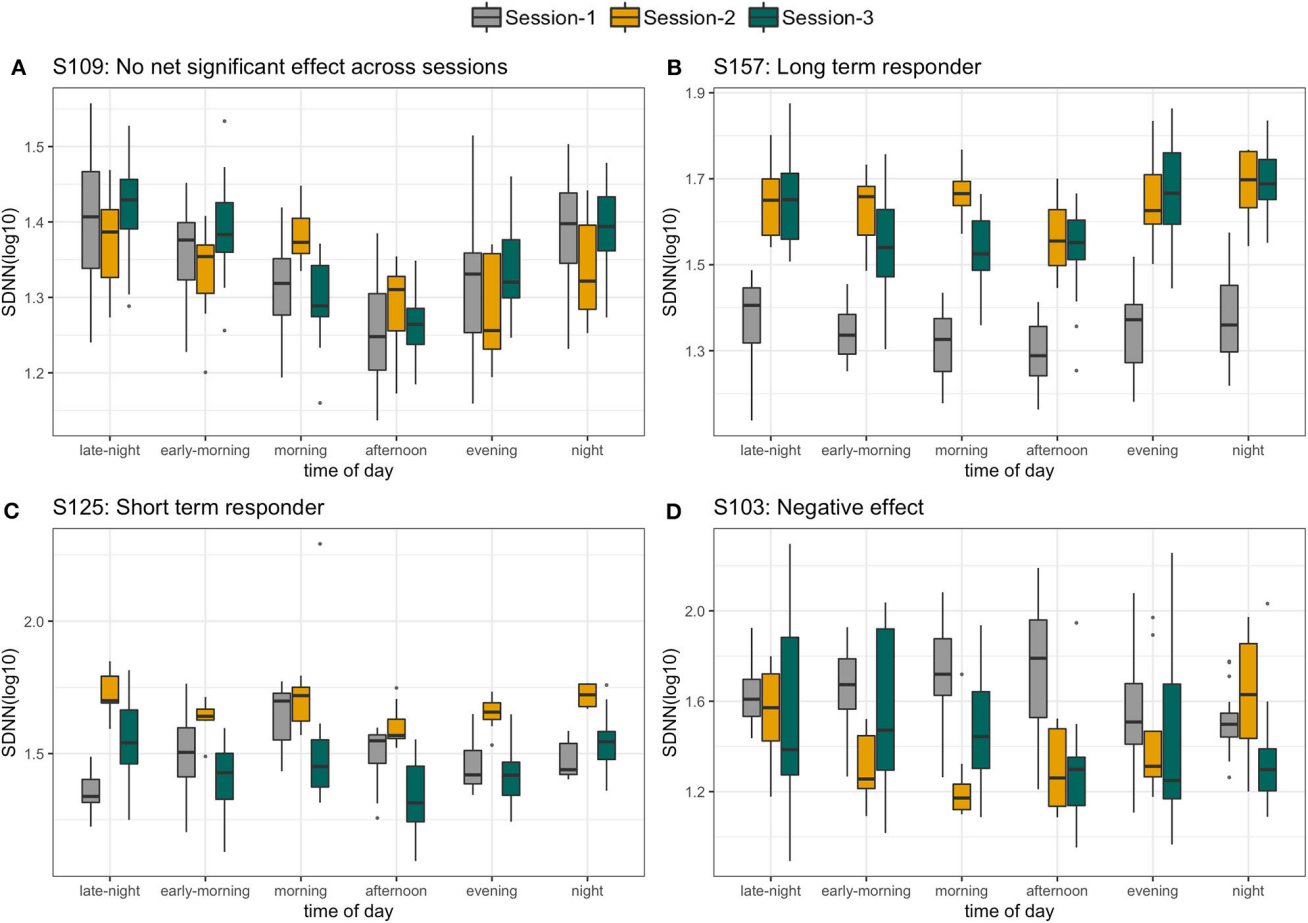
Four different profiles of individuals selected on the basis of personalized response to wellness center visit. **(A)** No significant difference in standard deviation of interbeat intervals (SDNN) across the three sessions. **(B)** Long-term responder, **(C)** short-term responder, and **(D)** persistent decrease in SDNN.

## Discussion

We sought to better understand changes in HRV over time and how it can be influenced, both in the short and longer term, by active participation in week-long wellness or vacation relaxation interventions. Key findings from our study show the potential utility of continual real-world monitoring of HRV to assess individualized response to health and wellness interventions including inter- and intraperson variability over time. Our findings provide further evidence of the value of utilizing personalized and longitudinal physiological monitoring approaches for assessing individualized health and wellness (“precision health”) (43).

The cohort level models showed the efficacy of HR- and HRV-related biosignals collected in real-world settings to be associated with known demographic and physiological characteristics such as age (44), BMI (45), and physical activity (46). While we did not find a significant and persistent difference in HRV response among those participating in the PH program vs. the regular vacation (RELAX), both groups showed a significant positive increase in HRV while being at a resort location. This confirms that a relaxing environment does, at least on a population level, lead to an overall general increase in parasympathetic activity due to, presumably, a decrease in stress. As has been previously described (41, 42), significant diurnal variations in HRV were also observed at the individual level that should be taken into consideration while analyzing longitudinal physiological data. This variation could also be associated with long-term health, manifested by nocturnal drops in blood pressure and pulse (47).

Through continuous monitoring, we identified individuals with varying degrees of HRV responses in the three different sessions. Some individuals (exemplified by participant S157) were found to have a significant increase in HRV during session 2, which was maintained through follow-up in session 3, supporting a relaxation response that was sustained (Figure 9B). Others, such as participant S125, were found to have an increase in HRV during session 2, but by session 3, both had returned to session 1 levels (Figure 9C). Finally, the two other cases were consistent with no relaxation or with both no significant HRV differences across the sessions (S109, Figure 9A) or with a significant decrease in HRV (S103, Figure 9D) in sessions 2 and 3 compared to session 1.

HRV has been suggested to be of value as an objective measure of an individual's level of stress or calmness, as it measures fluctuations in autonomic nervous system input to the heart and the changing balance between parasympathetic and sympathetic systems influences. With the growing availability of sensor technologies designed for personal use that are able to continuously track heart rate and HRV, this can provide people with valuable insights into their stress level, and potentially more importantly, an understanding of what activities impact that level, both positively or negatively. While prior studies have confirmed that HRV changes track well with acute emotional stress, such as when skydiving, its value in measuring long-term changes in calmness has been more difficult to study (48).

A wide range of personal digital health technologies (12) has recently made it much easier for interested individuals to measure their heart rate, either by using pulse rate measured via photoplethysmography (PPG), common to many smartwatches or other wearable devices, or by ECG. While both methods appear to be equally accurate for measuring heart rate, especially at rest, the very subtle, millisecond differences in measurements of beat-to-beat variation in heart rate are still more accurately assessed using an ECG (49). For an individual to track their own ECG, current devices available to consumers only allow for very short (usually 30 s) sampling of HR and HRV. Our results suggest that such instantaneous snapshots of HRV may not adequately capture important situational changes unless tracked at high frequency. In fact, much of the existing data on HRV and clinical outcomes are based on just 24 h of ECG recording. Until PPG sensors can be further refined, ECG-based wearables, especially multiday wear ECG patches as used in the current study, are likely to be most valuable in tracking daily and day-to-day fluctuations in HRV (50). Additionally, while salivary cortisol is frequently used as a biomarker of stress (51, 52), in the present sample, we did not find any significant changes in cortisol levels at the cohort level besides the known diurnal variations. This may further suggest the utility of high-frequency sensor-based objective assessment of physiological changes such as HRV over infrequent biological assays that may not be able to capture the dynamic response. Individualized differences in cortisol levels were not assessed due to limited data (one measurement per time of day) across sessions.

Most of what is currently known about HRV as a measure of risk in cardiovascular disease is based on population-level cutoffs of risk (23). Our findings suggest that with such high heterogeneity in HRV levels among individuals, a more meaningful measure of changes in autonomic activity would be based on deviations from individual-level baselines over time. In addition, external factors such as medications (53) and mood disorders (54) can impact an individual's HRV, making it even more important to track individualized responses. Heart rate recovery after exercise is another measure of autonomic nervous system input to the heart that is determined by recording the velocity of decrease in heart rate following exercise and has also been found to be associated with cardiovascular outcomes (55). Although it is highly personalized based on an individual's exercise capacity, the expected decrease in heart rate at 1 or 2 min after exertion is population based. In our study, HRV was correlated with activity during monitoring, but that might just have been a surrogate for individuals' levels of activity, and therefore cardiorespiratory fitness and vagal activity prior to participation. Novel personal wearable sensors (12)—including watches, rings, clothing, earphones, and more—that allow a person's unique changes in HR and HRV to be continuously monitored and benchmarked to themselves rather than a population norm may have great value in improving wellness and preventing both mental and cardiovascular disease. The findings support the viability of the continuous tracking of HRV to objectively measure an individual's response to potentially stressful or relaxing stimuli. Approaches to wellness, whether structured or unstructured, can support the restoration of HRV.

The present study also surfaces challenges in the use of wearable technology for longitudinal monitoring. Recent research has shown that participant retention (56) and compliance (57), i.e., the amount of time the wearable sensor patch is worn continuously, remains a challenge. Of the 112 enrolled participants, only 22 (19.6%) wore the patch for at least 24 h across all three sessions. The resulting data sparsity affected cohort and individual-level analyses, restricting the use of more generalized methods such as mixed effect models. However, despite the present data collection challenges, the use of wearable technology was shown to have a promising prospect for fully remote prospective intervention trials. The ability to objectively assess individualized physiological responses to interventions in a real-world setting at scale and at a fraction of the cost could help advance our ability to assess potential beneficial effects of a multiple of different types of wellness intervention programs.

## Conclusion

Simple, wearable sensors enable the ability to continuously monitor HRV during routine daily activities. Personalized HRV analysis at an individual level showed distinct HRV trajectories, further supporting the utility of using continuous real-world tracking of HRV at an individual level to objectively measure responses to potentially stressful or relaxing settings. This holds promise for identifying personalized stress and calm. However, there remain substantial challenges to fully understanding how to best measure it to extract actionable data to benefit the individual.

## Data Availability Statement

The datasets generated for this study are available on request to the corresponding author.

## Ethics Statement

The studies involving human participants were reviewed and approved by the UC San Diego Institutional Review Board (#171715) and registered on ClinicalTrials.gov with Identifier: NCT02241226. The patients/participants provided their written informed consent to participate in this study.

## Author Contributions

AP and EN: Data QA/QC, statistical analysis, preparation of the first manuscript draft, and major revisions. SS: study design, preparation of the first manuscript draft, and major revisions. SW: processed raw sensor-generated data to generate HRV features. CP: study design, study participant recruitment, testing, and manuscript editing. LW: study design, study participant enrollment, and testing, manuscript editing. SP: study design, study participant testing, and manuscript editing. DC: study design and manuscript editing. PM: study design, study participant testing, and manuscript editing. All authors contributed to the article and approved the submitted version.

## Conflict of Interest

SP is employed by the Chopra Global, New York. LW is employed by the Chopra Foundation. CP is a UC San Diego Post-Doctoral Fellow partially supported by the Chopra Foundation. DC is a co-founder of the Chopra Foundation. PM is the director of research at the Chopra Foundation. SW was employed by the company PhysIQ. The remaining authors declare that the research was conducted in the absence of any commercial or financial relationships that could be construed as a potential conflict of interest.

## References

[1] Schneiderman N Ironson G Siegel SD. Stress and health: psychological, behavioral, and biological determinants. Annu Rev Clin Psychol. (2005) 1:607–28. 10.1146/annurev.clinpsy.1.102803.144141 17716101 PMC2568977

[2] Golbidi S Frisbee JC Laher I. Chronic stress impacts the cardiovascular system: animal models and clinical outcomes. Am J Physiol Heart Circ Physiol. (2015) 308:H1476–98. 10.1152/ajpheart.00859.2014 25888514

[3] Janssen M Heerkens Y Kuijer W van der Heijden B Engels J. Effects of mindfulness-based stress reduction on employees' mental health: a systematic review. PLoS ONE. (2018) 13:e0191332. 10.1371/journal.pone.0191332 29364935 PMC5783379

[4] Lupien SJ McEwen BS Gunnar MR Heim C. Effects of stress throughout the lifespan on the brain, behaviour and cognition. Nat Rev Neurosci. (2009) 10:434–45. 10.1038/nrn2639 19401723

[5] Sliwinski MJ Almeida DM Smyth J Stawski RS. Intraindividual change and variability in daily stress processes: findings from two measurement-burst diary studies. Psychol Aging. (2009) 24:828–40. 10.1037/a0017925 20025399 PMC2857711

[6] Kim H-G Cheon E-J Bai D-S Lee YH Koo B-H. Stress and heart rate variability: a meta-analysis and review of the literature. Psychiatry Investig. (2018) 15:235–45. 10.30773/pi.2017.08.17 29486547 PMC5900369

[7] de Looff PC Cornet LJM Embregts PJCM Nijman HLI Didden HCM. Associations of sympathetic and parasympathetic activity in job stress and burnout: a systematic review. PLoS ONE. (2018) 13:e0205741. 10.1371/journal.pone.0205741 30335812 PMC6193670

[8] Smets E De Raedt W Van Hoof C. Into the wild: the challenges of physiological stress detection in laboratory and ambulatory settings. IEEE J Biomed Health Inform. (2019). 23, 463–73. 10.1109/JBHI.2018.2883751 30507517

[9] Léonard A Clément S Kuo C-D Manto M. Changes in heart rate variability during heartfulness meditation: a power spectral analysis including the residual spectrum. Front Cardiovasc Med. (2019) 6:62. 10.3389/fcvm.2019.00062 31139634 PMC6527777

[10] Shaffer F Ginsberg JP. An overview of heart rate variability metrics and norms. Front Public Health. (2017) 5:258. 10.3389/fpubh.2017.00258 29034226 PMC5624990

[11] Peake JM Kerr G Sullivan JP. A critical review of consumer wearables, mobile applications, and equipment for providing biofeedback, monitoring stress, and sleep in physically active populations. Front Physiol. (2018) 9:743. 10.3389/fphys.2018.00743 30002629 PMC6031746

[12] Dunn J Runge R Snyder M. Wearables and the medical revolution. Per Med. (2018) 15:429–448. 10.2217/pme-2018-0044 30259801 PMC12294383

[13] Jarow JP LaVange L Woodcock J. Multidimensional evidence generation and FDA regulatory decision making: defining and using “real-world” data. JAMA. (2017) 318:703–4. 10.1001/jama.2017.9991 28715550

[14] Perry B Herrington W Goldsack JC Grandinetti CA Vasisht KP Landray MJ. Use of mobile devices to measure outcomes in clinical research, 2010–2016: a systematic literature review. DIB. (2018) 2:11–30. 10.1159/000486347 29938250 PMC6008882

[15] Steinhubl SR Muse ED Topol EJ. The emerging field of mobile health. Sci Transl Med. (2015) 7:283rv3. 10.1126/scitranslmed.aaa3487 25877894 PMC4748838

[16] Buchheit M Simpson BM Garvican-Lewis LA Hammond K Kley M Schmidt WF. Wellness, fatigue and physical performance acclimatisation to a 2-week soccer camp at 3600 m (ISA3600). Br J Sports Med. (2013) 47 (Suppl. 1):i100–6. 10.1136/bjsports-2013-092749 24282195 PMC3903314

[17] Watt D Verma S Flynn L. Wellness programs: a review of the evidence. CMAJ. (1998) 158:224–30. 9469146 PMC1232698

[18] Moss D Biofeedback: a practitioner's guide (4th edition). In: Mark S, editor. Schwartz and Frank Andrasik. New York, NY: Guilford Press (2016). p. 764.

[19] Drury RL Porges S Thayer J Ginsberg JP. Editorial: Heart rate variability, health and well-being: a systems perspective. Front Public Health. (2019) 7:323. 10.3389/fpubh.2019.00323 31750285 PMC6848255

[20] Laborde S Mosley E Thayer JF. Heart rate variability and cardiac vagal tone in psychophysiological research - recommendations for experiment planning, data analysis, and data reporting. Front Psychol. (2017) 8:213. 10.3389/fpsyg.2017.00213 28265249 PMC5316555

[21] Tarvainen MP Niskanen J-P Lipponen JA Ranta-aho PO Karjalainen PA. Kubios HRV — a software for advanced heart rate variability analysis. In: IFMBE Proceedings. Antwerp (2009). p. 1022–5. 10.1007/978-3-540-89208-3_24324054542

[22] Tarvainen MP Niskanen J-P Lipponen JA Ranta-aho PO Karjalainen PA. Kubios HRV – Heart rate variability analysis software. In: Computer Methods and Programs in Biomedicine. Munich (2014). p. 210–20. 10.1016/j.cmpb.2013.07.024 24054542

[23] Heart rate variability Standards of measurement, physiological interpretation, and clinical use. Task Force of the European Society of Cardiology and the North American Society of Pacing and Electrophysiology. Eur Heart J. (1996). 17:354–81. 8737210

[24] Shaffer F McCraty R Zerr CL. A healthy heart is not a metronome: an integrative review of the heart's anatomy and heart rate variability. Front Psychol. (2014). 5:1040. 10.3389/fpsyg.2014.01040 25324790 PMC4179748

[25] Ryan R Booth S Spathis A Mollart S Clow A. Use of salivary diurnal cortisol as an outcome measure in randomised controlled trials: a systematic review. Ann Behav Med. (2016) 50:210–36. 10.1007/s12160-015-9753-9 27007274 PMC4823366

[26] Ho RTH Fong TCT Yip PSF. Perceived stress moderates the effects of a randomized trial of dance movement therapy on diurnal cortisol slopes in breast cancer patients. Psychoneuroendocrinology. (2018) 87:119–26. 10.1016/j.psyneuen.2017.10.012 29059542

[27] de Bloom J Geurts SAE Kompier MAJ. Effects of short vacations, vacation activities and experiences on employee health and well-being. Stress Health. (2012) 28:305–18. 10.1002/smi.1434 22213478

[28] Mills PJ Wilson KL Pung MA Weiss L Patel S Doraiswamy PM. The self-directed biological transformation initiative and well-being. J Altern Complement Med. (2016) 22:627–34. 10.1089/acm.2016.0002 27351443

[29] Cahn BR Goodman MS Peterson CT Maturi R Mills PJ. Yoga, meditation and mind-body health: increased bdnf, cortisol awakening response, and altered inflammatory marker expression after a 3-month yoga and meditation retreat. Front Hum Neurosci. (2017) 11:315. 10.3389/fnhum.2017.00315 28694775 PMC5483482

[30] Peterson CT Lucas J John-Williams LS Thompson JW Moseley MA Patel S. Corrigendum: identification of altered metabolomic profiles following a panchakarma-based ayurvedic intervention in healthy subjects: the self-directed biological transformation initiative (SBTI). Sci Rep. (2016) 6:34678. 10.1038/srep34678 27721380 PMC5056448

[31] Proprietary Personalized Analytics and Cloud-Based IT Platform In: PhysIQ. Available: https://www.physiq.com/technology/ (accessed December, 28 2018).

[32] Tan G Dao TK Farmer L Sutherland RJ Gevirtz R. Heart rate variability (HRV) and posttraumatic stress disorder (PTSD): a pilot study. Appl Psychophysiol Biofeedback. (2011) 36:27–35. 10.1007/s10484-010-9141-y 20680439

[33] Liang K-Y Zeger SL Longitudinal data analysis using generalized linear models. Biometrika. (1986) 13 10.1093/biomet/73.1.13

[34] Harrison XA Donaldson L Correa-Cano ME Evans J Fisher DN Goodwin CED. A brief introduction to mixed effects modelling and multi-model inference in ecology. PeerJ. (2018) 6:e4794. 10.7717/peerj.4794 29844961 PMC5970551

[35] Bolker BM Brooks ME Clark CJ Geange SW Poulsen JR Stevens MHH. Generalized linear mixed models: a practical guide for ecology and evolution. Seidlhofer B, editor. Trends Ecol Evol. (2009) 24:127–35. 10.1016/j.tree.2008.10.008 19185386

[36] Bates D Mächler M Bolker B Walker S Fitting linear mixed-effects models using lme4. J Stat Softw. (2015) 67 10.18637/jss.v067.i01

[37] Newey WK West KD A simple, positive semi-definite, heteroskedasticity and autocorrelation consistent covariance matrix. Econometrica. (1987) 55:703–8. 10.2307/1913610

[38] West K Newey W Automatic lag selection in covariance matrix estimation. Natl Bur Econ Res. (1995) 61:631–53. 10.3386/t0144

[39] Zeileis A Econometric computing with HC and HAC covariance matrix estimators. J Stat Softw. (2004) 11:1–17. 10.18637/jss.v011.i10

[40] R The R Project for Statistical Computing. Available online at: https://www.r-project.org/ (accessed February 3, 2019).

[41] Rose RM Kreuz LE Holaday JW Sulak KJ Johnson CE. Diurnal variation of plasma testosterone and cortisol. J Endocrinol. (1972) 54:177–8. 10.1677/joe.0.0540177 5046590

[42] Ice GH Katz-Stein A Himes J Kane RL. Diurnal cycles of salivary cortisol in older adults. Psychoneuroendocrinology. (2004) 29:355–70. 10.1016/S0306-4530(03)00034-9 14644066

[43] Schüssler-Fiorenza Rose SM Contrepois K Moneghetti KJ Zhou W Mishra T Mataraso S. A longitudinal big data approach for precision health. Nat Med. (2019) 25:792–804. 10.1038/s41591-019-0414-6 31068711 PMC6713274

[44] Junior EC Oliveira FM. Attenuation of vagal modulation with aging: univariate and bivariate analysis of HRV. Conf Proc IEEE Eng Med Biol Soc. (2017) 2017:3178–81. 10.1109/EMBC.2017.8037532 29060573

[45] Windham BG Fumagalli S Ble A Sollers JJ Thayer JF Najjar SS. The relationship between heart rate variability and adiposity differs for central and overall adiposity. J Obes. (2012) 2012:149516. 10.1155/2012/149516 22649714 PMC3357556

[46] Blom EH Olsson EMG Serlachius E Ericson M Ingvar M. Heart rate variability is related to self-reported physical activity in a healthy adolescent population. Eur J Appl Physiol. (2009)105:877–83. 10.1007/s00421-009-1089-3 19479275 PMC2718191

[47] Dauphinot V Gosse P Kossovsky MP Schott A-M Rouch I Pichot V. Autonomic nervous system activity is independently associated with the risk of shift in the non-dipper blood pressure pattern. Hypertens Res. (2010) 33:1032–7. 10.1038/hr.2010.130 20668452

[48] Dikecligil GN Mujica-Parodi LR. Ambulatory and challenge-associated heart rate variability measures predict cardiac responses to real-world acute emotional stress. Biol Psychiatry. (2010) 67:1185–90. 10.1016/j.biopsych.2010.02.001 20299007 PMC2882500

[49] Schäfer A Vagedes J. How accurate is pulse rate variability as an estimate of heart rate variability? A review on studies comparing photoplethysmographic technology with an electrocardiogram. Int J Cardiol. (2013) 166:15–29. 10.1016/j.ijcard.2012.03.119 22809539

[50] Dobbs WC Fedewa MV MacDonald HV Holmes CJ Cicone ZS Plews DJ. The accuracy of acquiring heart rate variability from portable devices: a systematic review and meta-analysis. Sports Med. (2019) 49:417–35. 10.1007/s40279-019-01061-5 30706234

[51] Bozovic D Racic M Ivkovic N. Salivary cortisol levels as a biological marker of stress reaction. Mediev Archaeol. (2013) 67:374–7. 10.5455/medarh.2013.67.374-377 24601177

[52] Hellhammer DH Wüst S Kudielka BM. Salivary cortisol as a biomarker in stress research. Psychoneuroendocrinology. (2009) 34:163–71. 10.1016/j.psyneuen.2008.10.026 19095358

[53] Garakani A Martinez JM Aaronson CJ Voustianiouk A Kaufmann H Gorman JM. Effect of medication and psychotherapy on heart rate variability in panic disorder. Depress Anxiety. (2009) 26:251–8. 10.1002/da.20533 18839407

[54] Hughes JW Stoney CM. Depressed mood is related to high-frequency heart rate variability during stressors. Psychosom Med. (2000) 62:796–803. 10.1097/00006842-200011000-00009 11138999

[55] Cole CR Blackstone EH Pashkow FJ Snader CE Lauer MS. Heart-rate recovery immediately after exercise as a predictor of mortality. N Engl J Med. (1999) 341:1351–7. 10.1056/NEJM199910283411804 10536127

[56] Pratap A Neto EC Snyder P Stepnowsky C Elhadad N Grant D. Indicators of retention in remote digital health studies: a cross-study evaluation of 100,000 participants. NPJ Digit Med. (2020) 3:21. 10.1038/s41746-020-0224-8 32128451 PMC7026051

[57] Waalen J Peters M Ranamukhaarachchi D Li J Ebner G Senkowsky J. Real world usage characteristics of a novel mobile health self-monitoring device: results from the Scanadu Consumer Health Outcomes (SCOUT) Study. PLoS ONE. (2019) 14:e0215468. 10.1371/journal.pone.0215468 30990860 PMC6467418

