# Supplementary material for: Changes in Continuous, Long-Term Heart Rate Variability and Individualized Physiological Responses to Wellness and Vacation Interventions Using a Wearable Sensor
Source: Front Cardiovasc Med. 2020 Jul 31;7:120. doi: 10.3389/fcvm.2020.00120 (PMC7411743; doi:10.3389/fcvm.2020.00120)
Supplement: Supplementary file 1 [file Data_Sheet_1.PDF]

Supplementary Table 1: Comparison of participant demographics across three groups that is randomly allocated to i) PH intervention, ii) RELAX intervention and iii) non-random assignment to PH intervention

	**PH**	**PH (non random)**	**RELAX**	**p-value**
N	50	19	44	
Age (mean (SD))	53.58 (11.85)	58.32 (12.76)	54.80 (11.41)	0.348
Gender = Female (%)	35 (77.8)	14 (73.7)	27 (75.0)	0.926
Height (mean (SD))	65.85 (3.31)	67.25 (3.35)	66.03 (3.88)	0.341
Weight (mean (SD))	149.75 (34.03)	147.42 (27.15)	154.14 (39.71)	0.761

Supplementary Table 2: Cohort level GEE model - HRV Features

	HR	HRV	log10(AHF)	log10(ALF)	log10(LF/HF)	log10(PLF)	log10(PHF)
(Intercept)	153.44 (7.79)***	53.98 (14.33)***	-3.57 (0.29)***	-2.19 (0.24)***	1.70 (0.20)***	-0.55 (0.23)*	-1.92 (0.31)***
Age	-0.12 (0.06)·	-0.36 (0.14)*	-0.02 (0.00)***	-0.01 (0.00)***	0.00 (0.00)·	-0.01 (0.00)**	-0.01 (0.00)**
Gender:Male	-5.61 (1.60)**	-1.46 (3.15)	-0.16 (0.07)*	-0.01 (0.07)	0.14 (0.04)**	0.02 (0.04)	-0.12 (0.04)**
BMI	0.31 (0.15)	-0.49 (0.27)·	-0.01 (0.01)	-0.01 (0.01)·	0.00 (0.00)	-0.01 (0.00)***	-0.01 (0.00)
**Time of Day**							
late-night	13.60 (5.40)*	-4.35 (9.13)	-0.02 (0.12)	-0.13 (0.13)	0.02 (0.12)	-0.85 (0.21)***	-0.71 (0.25)*
morning	2.42 (4.87)	-1.13 (7.79)	0.20 (0.15)	0.20 (0.14)	0.07 (0.12)	0.35 (0.26)	0.36 (0.27)
afternoon	5.33 (5.81)	7.12 (10.97)	-0.00 (0.22)	0.16 (0.17)	0.37 (0.17)*	1.02 (0.33)*	0.91 (0.39)
evening	29.60 (8.29)**	12.74 (17.50)	-0.03 (0.27)	-0.13 (0.28)	0.33 (0.21)	0.55 (0.31)	0.50 (0.38)
night	38.21 (8.17)***	-10.03 (12.88)	-0.51 (0.29)	-0.38 (0.23)	0.71 (0.19)***	0.56 (0.25)	0.52 (0.32)
**Cohort:**PH	-0.81 (2.23)	-0.66 (3.44)	-0.07 (0.09)	-0.09 (0.07)	0.00 (0.05)	-0.02 (0.06)	-0.01 (0.08)
**Session**							
Session-2	-0.59 (1.55)	-0.48 (2.88)	-0.01 (0.06)	-0.08 (0.03)*	-0.05 (0.05)	-0.06 (0.06)	0.04 (0.07)
Session-3	1.50 (1.61)	1.93 (2.64)	0.03 (0.07)	-0.07 (0.05)	-0.07 (0.04)	0.03 (0.05)	0.12 (0.07)
**Physical Activity x Time of Day**							
ACT:late-night	8.92 (3.00)*	-4.38 (5.30)	-0.04 (0.07)	-0.07 (0.07)	0.04 (0.07)	-0.42 (0.12)***	-0.37 (0.14)*
ACT:morning	1.42 (2.80)	-0.45 (4.55)	0.13 (0.08)	0.15 (0.08)·	0.06 (0.07)	0.17 (0.14)	0.17 (0.15)
ACT:afternoon	3.53 (3.05)	4.77 (6.07)	0.01 (0.12)	0.14 (0.10)	0.25 (0.09)*	0.60 (0.18)**	0.51 (0.21)*
ACT:evening	17.41 (4.34)***	5.54 (9.34)	-0.06 (0.14)	-0.04 (0.15)	0.25 (0.12)	0.38 (0.17)	0.30 (0.21)
ACT:night	23.18 (4.33)***	-7.29 (7.07)	-0.33 (0.15)*	-0.20 (0.13)	0.43 (0.10)***	0.37 (0.13)*	0.31 (0.17)
**Cohort x Session**							
CohortPH:Session-2	-0.24 (2.29)	-0.60 (3.81)	-0.06 (0.09)	0.04 (0.06)	0.09 (0.06)	-0.00 (0.08)	-0.14 (0.10)
CohortPH:Session-3	-2.59 (2.29)	-0.37 (3.39)	0.00 (0.09)	0.04 (0.06)	0.00 (0.06)	0.05 (0.07)	0.01 (0.09)
**Cohort x Time of Day**							
late-night:CohortPH	-0.86 (1.51)	-0.01 (2.03)	0.01 (0.04)	0.06 (0.04)	0.03 (0.03)	0.05 (0.06)	0.02 (0.06)
morning:CohortPH	0.51 (1.33)	-0.39 (1.69)	0.01 (0.05)	0.03 (0.03)	0.01 (0.03)	0.00 (0.06)	-0.00 (0.07)
afternoon:CohortPH	1.33 (1.76)	0.57 (2.39)	0.01 (0.06)	0.06 (0.05)	0.04 (0.05)	0.02 (0.05)	-0.01 (0.06)
evening:CohortPH	-0.24 (2.14)	-2.48 (2.91)	-0.05 (0.09)	0.05 (0.05)	0.09 (0.06)	0.11 (0.07)	0.05 (0.08)
night:CohortPH	-0.33 (2.12)	-0.82 (3.50)	-0.01 (0.09)	0.07 (0.06)	0.05 (0.06)	0.07 (0.07)	0.03 (0.08)

**Session x Time of Day**							
late-night:Session-2	1.80 (1.47)	-2.32 (2.68)	-0.09 (0.07)	-0.02 (0.05)	0.07 (0.05)	0.12 (0.11)	-0.02 (0.09)
morning:Session-2	-2.37 (1.70)	3.08 (3.29)	0.16 (0.05)**	0.17 (0.04)***	0.01 (0.03)	0.11 (0.06)·	0.08 (0.07)
afternoon:Session-2	-4.26 (1.69)*	6.77 (2.95)*	0.15 (0.07)*	0.19 (0.05)**	0.05 (0.05)	0.12 (0.05)*	0.07 (0.06)
evening:Session-2	-2.10 (2.26)	-0.01 (4.35)	0.01 (0.10)	0.10 (0.06)	0.07 (0.09)	0.10 (0.08)	-0.01 (0.09)
night:Session-2	0.68 (2.76)	0.35 (3.47)	-0.07 (0.12)	0.02 (0.07)	0.08 (0.08)	0.17 (0.09)	0.05 (0.09)
late-night:Session-3	-2.10 (1.55)	2.69 (2.34)	0.03 (0.04)	0.11 (0.04)*	0.05 (0.03)	0.04 (0.06)	-0.03 (0.06)
morning:Session-3	-0.68 (1.47)	-1.23 (2.10)	-0.02 (0.05)	0.04 (0.03)	0.05 (0.04)	-0.00 (0.06)	-0.07 (0.07)
afternoon:Session-3	-2.25 (1.98)	-1.44 (2.35)	-0.03 (0.07)	0.07 (0.04)	0.10 (0.06)	-0.07 (0.06)	-0.18 (0.08)*
evening:Session-3	-2.88 (2.43)	-0.28 (2.63)	-0.05 (0.09)	0.11 (0.05)*	0.17 (0.07)*	0.14 (0.07)·	0.01 (0.09)
night:Session-3	-2.75 (2.26)	0.43 (3.58)	-0.02 (0.08)	0.12 (0.06)	0.12 (0.06)	0.03 (0.08)	-0.09 (0.09)
**Cohort x Session x Time of Day**							
late-night:CohortPH:Session-2	-0.89 (1.63)	1.28 (3.36)	0.09 (0.08)	0.01 (0.06)	-0.06 (0.05)	-0.07 (0.12)	0.09 (0.10)
morning:CohortPH:Session-2	0.33 (1.94)	0.11 (3.63)	-0.08 (0.07)	-0.13 (0.05)	-0.04 (0.04)	-0.09 (0.08)	-0.02 (0.09)
afternoon:CohortPH:Session-2	0.33 (1.96)	-4.21 (3.60)	-0.05 (0.08)	-0.14 (0.06)	-0.09 (0.06)	-0.18 (0.06)*	-0.10 (0.07)
evening:CohortPH:Session-2	-0.87 (2.57)	4.13 (5.05)	0.09 (0.12)	-0.05 (0.07)	-0.12 (0.10)	-0.21 (0.10)	-0.08 (0.12)
night:CohortPH:Session-2	-1.67 (2.87)	4.53 (4.55)	0.14 (0.13)	0.03 (0.08)	-0.08 (0.08)	-0.20 (0.11)	-0.05 (0.12)
late-night:CohortPH:Session-3	3.08 (1.77)·	-2.34 (2.93)	-0.04 (0.05)	-0.10 (0.05)	-0.02 (0.04)	-0.14 (0.08)	-0.10 (0.08)
morning:CohortPH:Session-3	-0.24 (1.67)	2.18 (2.52)	0.03 (0.06)	-0.02 (0.04)	-0.03 (0.04)	-0.01 (0.07)	0.03 (0.08)
afternoon:CohortPH:Session-3	1.31 (2.26)	0.55 (3.20)	0.00 (0.08)	-0.08 (0.06)	-0.06 (0.07)	-0.01 (0.07)	0.08 (0.09)
evening:CohortPH:Session-3	1.95 (2.69)	0.41 (3.55)	0.03 (0.10)	-0.09 (0.06)	-0.11 (0.08)	-0.25 (0.09)*	-0.18 (0.11)
night:CohortPH:Session-3	1.90 (2.49)	0.32 (4.32)	0.05 (0.10)	-0.09 (0.07)	-0.09 (0.07)	-0.22 (0.10)	-0.11 (0.10)
***p < 0.001, **p < 0.01, *p < 0.05							

Supplementary Table 3: Cohort level GEE model - Salivary cortisol levels

	Cortisol Levels
(Intercept)	5.57 (2.68)*
Cohort- RELAX	0.11 (0.55)
**Context**	
home month after	-0.45 (0.37)
resort week end	0.48 (0.31)
resort week start	0.15 (0.35)
**Time of Day: Ref (wake up time)**	
30min after waking up	0.67 (0.39)
12:00 PM	-1.29 (0.38)***
430PM	-2.16 (0.33)***
800PM	-2.15 (0.40)***
BEDTIME	-1.77 (0.35)***
Age	-0.01 (0.01)
Gender = Female	-0.17 (0.31)
Height	-0.00 (0.04)
Weight	-0.01 (0.00)
**Cohort x Context**	
RELAX:home month after	0.25 (0.69)
RELAX:resort week end	-0.81 (0.60)
RELAX:resort week start	-0.95 (0.63)
**Cohort x Time of Day**	
RELAX:30min	0.43 (0.60)
RELAX:12PM	0.15 (0.61)
RELAX:430PM	-0.41 (0.65)
RELAX:800PM	-0.32 (0.77)
RELAX:BEDTIME	0.66 (0.68)
**Context x Time of Day**	
home month after:30min	0.88 (0.50)
resort week end:30min	0.32 (0.48)
resort week start:30min	-0.32 (0.53)
home month after:12PM	0.12 (0.54)
resort week end:12PM	-0.76 (0.42)
resort week start:12PM	-0.91 (0.47)
home month after:430PM	0.57 (0.44)
resort week end:430PM	-0.44 (0.40)

resort week start:430PM	-0.40 (0.39)
home month after:800PM	0.45 (0.42)
resort week end:800PM	-0.02 (0.51)
resort week start:800PM	-0.76 (0.49)
home month after:BEDTIME	0.27 (0.45)
resort week end:BEDTIME	-0.41 (0.37)
resort week start:BEDTIME	-0.54 (0.47)
**Cohort x Context x Time of Day**	
RELAX:home month after:30min	-1.09 (0.95)
RELAX:resort week end:30min	0.34 (0.84)
RELAX:resort week start:30min	0.88 (0.80)
RELAX:home month after:12PM	-0.99 (0.91)
RELAX:resort week end:12PM	0.42 (0.74)
RELAX:resort week start:12PM	0.52 (0.74)
RELAX:home month after:430PM	-0.16 (1.02)
RELAX:resort week end:430PM	1.25 (0.81)
RELAX:resort week start:430PM	0.61 (0.75)
RELAX:home month after:800PM	-0.57 (0.91)
RELAX:resort week end:800PM	0.21 (0.87)
RELAX:resort week start:800PM	1.35 (0.93)
RELAX:home month after:BEDTIME	-1.31 (0.89)
RELAX:resort week end:BEDTIME	-0.30 (0.82)
RELAX:resort week start:BEDTIME	0.42 (0.90)
Scale parameter: gamma	3.23
Scale parameter: SE	0.21
Correlation parameter: alpha	0.45
Correlation parameter: SE	0.04
Num. obs.	1690
Num. clust.	97
***p < 0.001, **p < 0.01, *p < 0.05	

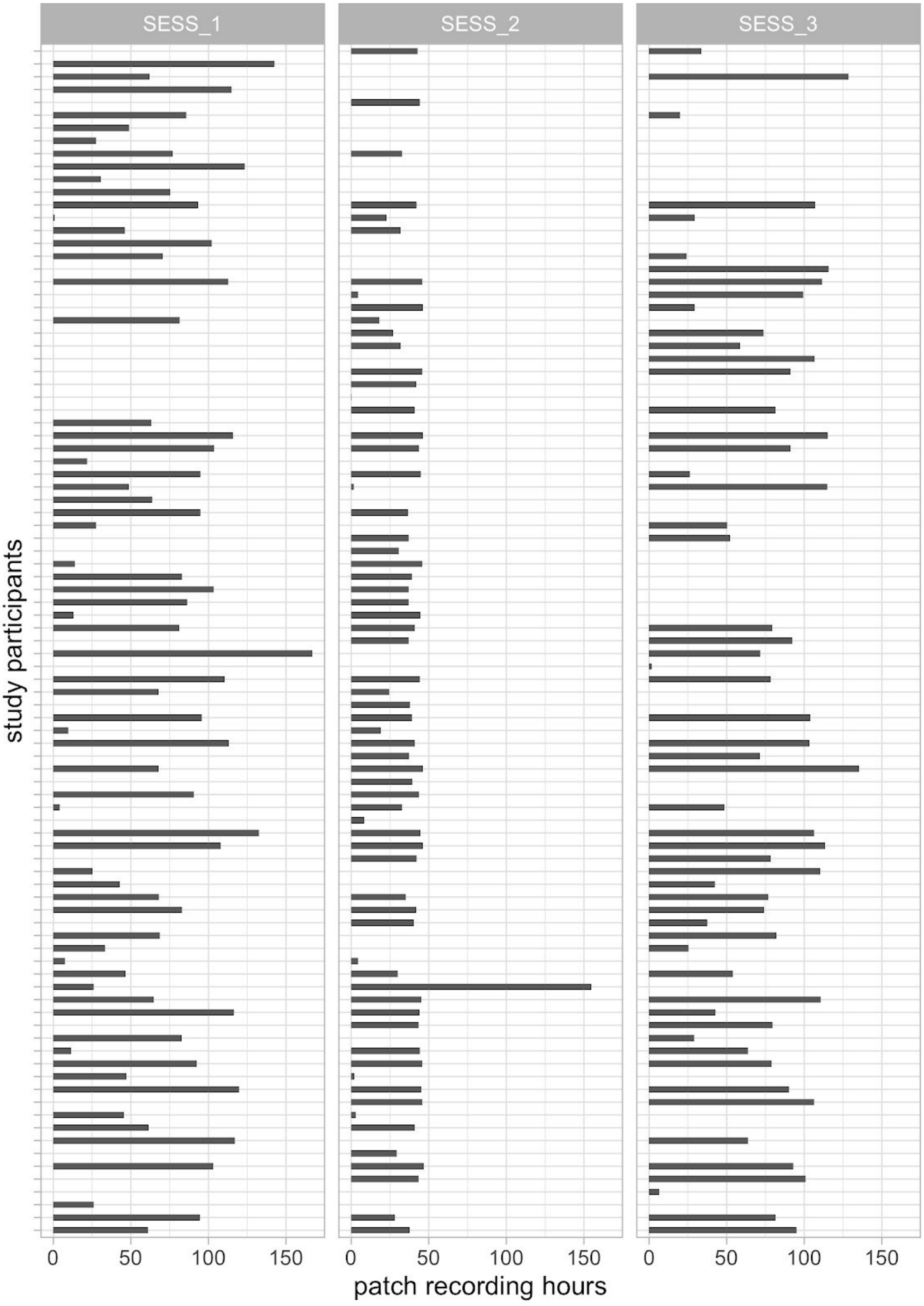

Supplementary Figure 1: Hours of recording per participant across the three sessions

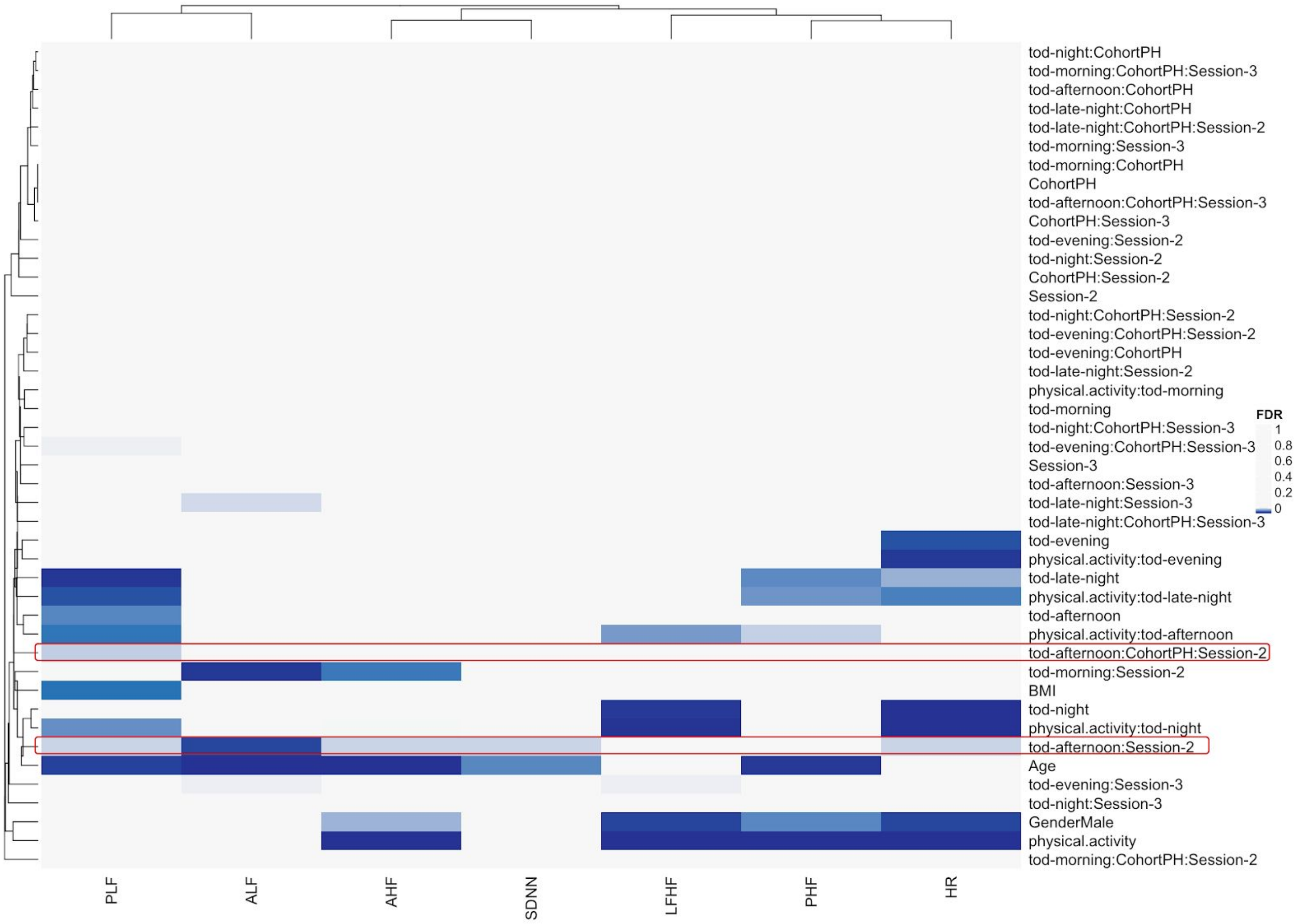

Supplementary Figure 2: Heatmap of showing statistically significant covariates(FDR corrected p-values) along from 7 different cohort level GEE models (one per each HRV related feature on the x-axis)

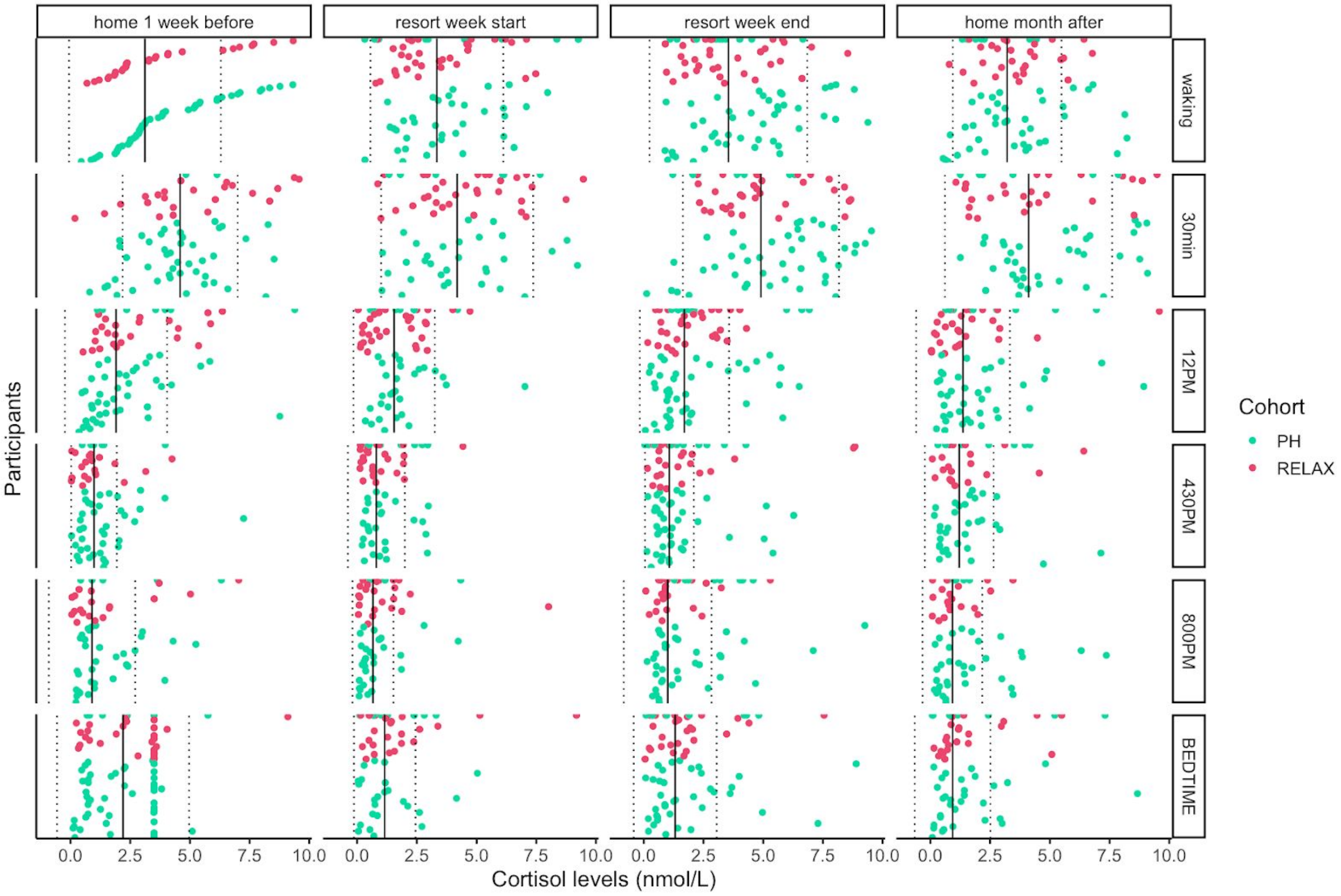

Supplementary Figure 3: Cortisol levels(nmol/L) across study participants faceted by time of day and location(x-axis). In each panel the solid black line is the median value of the sample with the dashed lines indicating the IQR range.

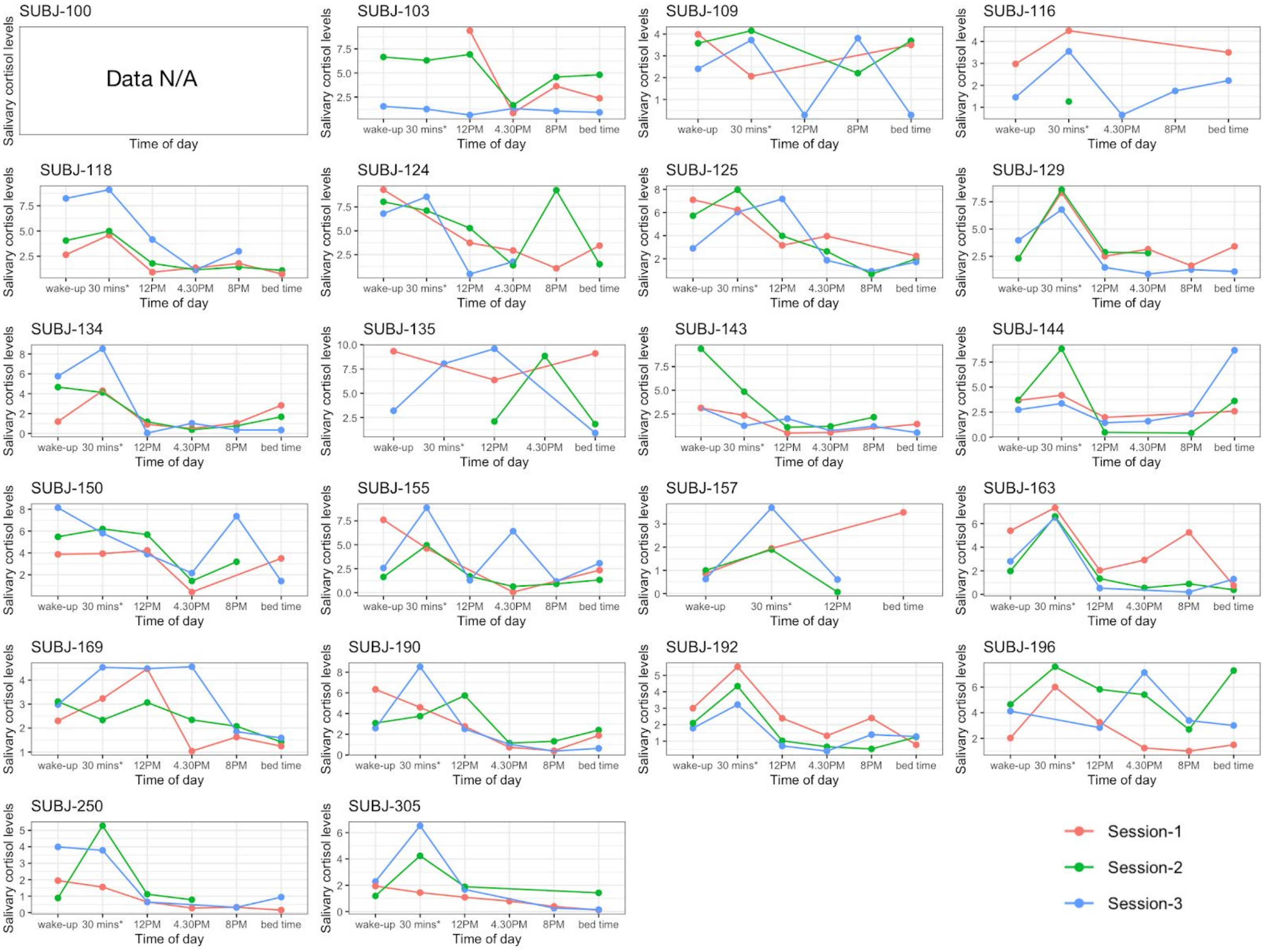

Supplementary Figure 4: Cortisol levels(nmol/L) per study participants across various times of day stratified by session.
